# Comparison of methods for inframammary fold fixation of deep inferior epigastric perforator flap breast reconstruction^✰^

**DOI:** 10.1016/j.jpra.2026.01.020

**Published:** 2026-01-23

**Authors:** Tomohiro Kojimahara, Hiroki Mori, Sayuri Kato, Kyoichi Murakami, Nanami Hamasaki, Koji Kanayama, Noriko Uemura, Kentaro Tanaka

**Affiliations:** a Plastic and Reconstructive Surgery, Institute of Science Tokyo, Tokyo, Japan; b Reconstructive Plastic Surgery, Institute of Science Tokyo, Tokyo, Japan

**Keywords:** Breast reconstruction, DIEP flap, Microsurgery, Perforator flap, Inframammary fold

## Abstract

**Background:**

Various methods for inframammary fold (IMF) fixation in deep inferior epigastric artery perforator (DIEP) flap breast reconstruction have been reported. This quantitative study focused on the comparison of IMF fixation results between immediate one-stage (internal fixation), delayed one-stage (de-epithelialized method), and two-stage (capsule fixation) reconstruction.

**Methods:**

A retrospective study of 34 patients who underwent unilateral DIEP flap reconstruction was conducted. The IMF height, grouped by reconstruction methods, was measured from the standardized clinical photographs >6 months after the DIEP flap reconstruction (post-DIEP) and >6 months after the flap revision surgery (final), and compared with the healthy side.

**Results:**

In the order of immediate one-stage, delayed one-stage, and two-stage reconstruction, respectively, the average (± standard deviation) post-DIEP IMF heights were −1.60 ± 0.65 cm, −0.19 ± 1.16 cm, and −1.40 ± 1.11 cm, and the average final IMF heights were −1.20 ± 0.44 cm, −0.19 ± 0.52 cm, and −0.68 ± 0.98 cm.

**Conclusion:**

After DIEP flap breast reconstruction, the IMF of the reconstructed side tends to be lower than the healthy side. The de-epithelialized method used in delayed one-stage reconstruction showed relatively good IMF fixation after DIEP flap reconstruction compared with other reconstruction methods. By performing additional IMF procedures during flap revision surgery, the average IMF height ranged from −1.2 to −0.19 cm compared with the healthy side.

## Introduction

Deep inferior epigastric artery perforator (DIEP) flap breast reconstruction is the current standard for autologous tissue breast reconstruction. This technique can be applied to relatively large defects and is able to recreate the ptosis of the natural breast. The inframammary fold (IMF) is an anatomical structure at the inferior pole of the breast that plays an important role in the left-right symmetry of the breast.^1^ Preservation of the IMF structure during mastectomy is well known to be important for better aesthetic results,^2^, ^3^, ^4^ and complete preservation is difficult. Various methods for IMF recreation have been reported, including local tissue rearrangement,^5^, ^6^, ^7^, ^8^, ^9^ reconstruction by suture techniques,^10^, ^11^, ^12^ and utilization of devices.^13^, ^14^, ^15^ However, most studies have focused on a single method of IMF redefinition. We have used different IMF fixation methods in DIEP flap reconstruction. We have used internal fixation^7^ for immediate one-stage reconstruction, the de-epithelialized method^9^ for delayed one-stage reconstruction, and capsule fixation^8^ for two-stage reconstruction. The present study aimed to compare IMF reconstruction procedures using quantitative evaluations among groups based on the reconstructive methods applied.

## Material and methods

Cases of unilateral DIEP flap breast reconstruction mainly performed by a single surgeon in the Department of Plastic and Reconstructive Surgery at the Institute of Science Tokyo Hospital between February 2018 and April 2023 were included in this retrospective study.

**Figure 1:**
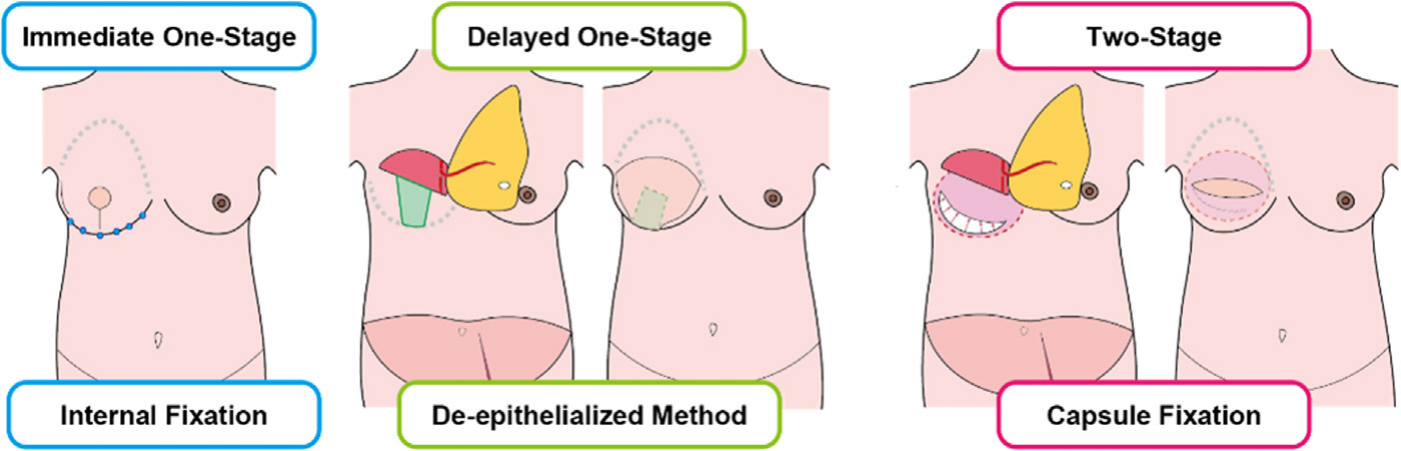
IMF procedures for reconstruction methods. Internal fixation was used in immediate one-stage reconstruction, the de-epithelialized method was used in delayed one-stage reconstruction, and capsule fixation was used in two-stage reconstruction.

Reconstruction procedures for the IMF varied according to the reconstruction method applied. In immediate one-stage reconstruction; DIEP flap breast reconstruction and mastectomy is performed in the same surgery; the loosened IMF was redefined by subcutaneous suture to the chest wall.^7^ In delayed one-stage reconstruction; DIEP flap breast reconstruction was performed on post-mastectomy patients with no prior reconstruction, the central area was de-epithelialized and left to the chest wall, the remaining lateral and medial part of the not de-epithelialized mastectomy flap functioned as a supportive hammock to support the DIEP flap.^9^ In two-stage reconstruction; tissue expander was inserted during the mastectomy, and the tissue expander was exchanged into autologous tissue in the DIEP flap breast reconstruction, as the subpectoral tissue expander was removed, a pre-pectoral space was made, and the capsule was repaired or excised.^8^ In this capsule repair, the capsule created by the latero-inferior part of the serratus anterior muscle’s fascia or external oblique muscle’s fascia was excised, each end was simply sutured with 3–0 polydioxanone with no reattachment to the costal perichondrium. For asymmetric cases, extra IMF fixation was performed in the flap revision surgery (Figure 1).

Clinical pictures standardized by CasMatch (Bear Medic, Yushima, Tokyo, Japan) were used for measurement. Perpendicular lines were drawn from the anterior midline to the caudal-most point of each IMF, then the difference in IMF heights between sides was assessed on ImageJ (National Institutes of Health, USA) in 5-mm increments. If the reconstructed side was elevated compared to the healthy side, the height was reported as a positive value. IMF measurements were conducted before flap revision and reconstruction of the nipple-areola complex (NAC), >6 months after DIEP flap reconstruction (post-DIEP), and >6 months after the final IMF procedure in flap revision and NAC reconstruction (final).

In cases where measurement was difficult, the IMF was measured by the underwear line. Exclusion criteria were cases in which it was impossible to measure the IMF due to severe breast ptosis, lack of clinical photos of post-DIEP or final conditions, or in which IMF fixation was not performed in the DIEP flap reconstruction surgery.

The manuscript was checked against the Strengthening the Reporting of Observational Studies in Epidemiology (STROBE) checklist (Supplemental Appendix).

### Statistics

Continuous variables were analyzed using the Wilcoxon signed-rank test (non-parametric value for paired groups) or Kruskal–Wallis test (non-parametric value for multiple groups), Dunn’s test was used for post-hoc analysis. Fisher’s exact test or chi-squared test was used for analysis of distribution statistics. Values of *p* < 0.05 were considered significant. All data were statistically analyzed using GraphPad Prism (version 10; GraphPad Software, San Diego, CA).

## Results

### Study population

Among the 95 unilateral DIEP flap reconstructions performed between February 2018 and April 2023, 48 cases lacked clinical pictures of the post-DIEP and/or final condition. Among the remaining 47 cases, IMF fixation was not performed in either DIEP flap reconstruction or flap revision surgery in 13 cases. The remaining 34 cases were included for analysis in this study: five cases in immediate one-stage reconstruction group, 13 cases in delayed one-stage reconstruction group, and 16 cases in two-stage reconstruction. No significant differences in average age, body mass index, radiation status, or flap weight for reconstruction were identified between patients grouped according to reconstruction method. For surgical excision methods, conventional total mastectomy was most frequently observed with delayed one-stage (10 cases) and two-stage reconstruction (nine cases). Seven patients received radiation therapy prior to DIEP flap reconstruction, and one patient received radiation therapy after the DIEP flap reconstruction and during the observation period.

The IMF procedures for DIEP reconstruction were internal fixation to the chest wall with immediate one-stage reconstruction in five cases. The de-epithelialized method was utilized in all cases of delayed one-stage reconstruction. Tissue expander capsule repair was used to create the IMF in two-stage reconstruction in all cases. In all cases, 3–0 polydioxanone was used as a fixation thread.

All cases with immediate one-stage reconstruction required an extra internal fixation, compared to six cases with delayed one-stage reconstruction and 10 cases with two-stage reconstruction. Among the delayed one-stage reconstruction IMF procedure in flap revision surgery described as other in Table 1, dissection of the subcutaneous plane was done in one case and skin excision to create IMF was performed in two cases. Four cases with delayed one-stage reconstruction and three cases with two-stage reconstruction did not require an extra IMF fixation. Three cases in immediate one-stage reconstruction, one case in delayed one-stage reconstruction and three cases in two-stage reconstruction did not require flap revision and the patient did not pursue NAC reconstruction. Among the remaining cases, average (± standard deviation) days of flap revision and NAC reconstruction were 289 ± 38.1 days (*n* = 5) in immediate one-stage reconstruction, 428 ± 192 days (*n* = 12) in delayed one-stage reconstruction and 249 ± 62.7 days (*n* = 13) in two-stage reconstruction. Average days for final measurement were 585 ± 639 days in immediate one-stage reconstruction, 446 ± 180 days in delayed one-stage reconstruction and 462 ± 360 days in two-stage reconstruction. However, no significant difference was observed among the number of procedures for IMF reconstruction (Table 1). None of the patients required fixation of the native breast.

**Table 1:** Patient characteristics.

	Internal fixation (Immediate one-stage) (*n* = 5)	De-epithelialized method (Delayed one-stage) (*n* = 13)	Capsule fixation (Two-stage) (*n* = 16)	*p*
Age [years]	52.4 (SD 8.7)	48.6 (SD 5.1)	50.6 (SD 9.0)	0.79
Surgery side				0.31
Right	4	8	7	
Left	1	5	9	
BMI [kg/m^2^]	23.0 (SD 3.0)	24.3 (SD 5.2)	23.3 (SD 3.3)	0.94
Flap weight for reconstruction [g]	585 (SD 220) (*n* = 4)	564 (SD 161) (*n* = 10)	510 (SD 230) (*n* = 13)	0.47
Mastectomy procedure				0.004
Total mastectomy	0	10	9	
SSM	5	1	7	
NSM	0	2	0	
Radiation Therapy				0.052
Before DIEP	0	6	1	
After DIEP	0	0	1	
No Radiation	5	7	14	
IMF fixation method in flap revision surgery				0.07
Internal fixation	5	6	10	
Capsule repair	0	0	3	
Other	0	3	0	
None	0	4	3	
Average Days of Flap Revision Surgery from DIEP flap reconstruction	289 (SD 38.1) (*n* = 5)	428 (SD 192) (*n* = 12)	249 (SD 62.7) (*n* = 13)	0.0049
Average Days of Final Measurement	585 (SD 639)	446 (SD 180)	462 (SD 360)	0.60
Procedures required for IMF fixation [times]	2 (SD 0.00)	1.69 (SD 0.48)	1.81 (SD 0.40)	0.35

Data are shown as mean (standard deviation [SD]) Abbreviations: BMI, body mass index; SSM, skin-sparing mastectomy; NSM, nipple-sparing mastectomy; IMF, inframammary fold; DIEP, deep inferior epigastric artery perforator.

(Kruskal–Wallis test, Chi-squared test).

### Comparison of IMF height at each stage

**Figure 2:**
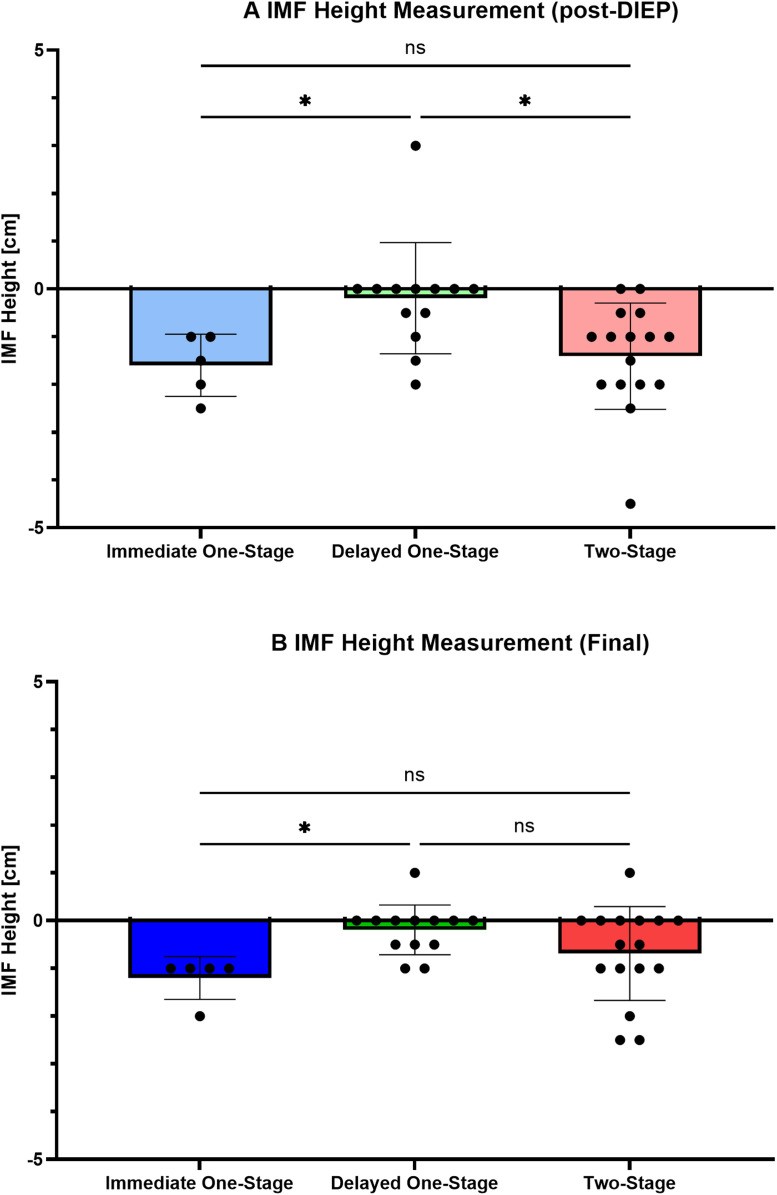
Comparison of IMF heights at each stage. **A)** Results at >6 months after DIEP flap reconstruction (post-DIEP). Immediate one-stage vs. Delayed one-stage: *p* = 0.022; Delayed one-stage vs. Two-stage: *p* = 0.015. **B)** Results at >6 months after final procedure of IMF. Immediate one-stage vs. Delayed one-stage: *p* = 0.019. Kruskal–Wallis test; *p < 0.05, **p < 0.01, ***p < 0.001, ns, not significant.

The height of the IMF compared to the healthy side was measured. Average height post-DIEP was −1.60 ± 0.65 cm for immediate one-stage (*n* = 5), −0.19 ± 1.16 cm for delayed one-stage (*n* = 13), and −1.40 ± 1.11 cm for two-stage (*n* = 16) (*p* = 0.0047). According to the multiple comparison, IMF was significantly lower with immediate one-stage reconstruction compared with delayed one-stage reconstruction (*p* = 0.022). IMF was also significantly lower with two-stage reconstruction than with delayed one-stage reconstruction (*p* = 0.015) (Figure 2A). Average height in the final condition was −1.20 ± 0.44 cm with immediate one-stage (*n* = 5), −0.19 ± 0.52 cm with delayed one-stage (*n* = 13), and −0.68 ± 0.98 cm with two-stage (*n* = 16) (*p* = 0.022). The final IMF was significantly lower with immediate one-stage reconstruction than with delayed one-stage reconstruction (*p* = 0.019) (Figure 2B).

### Change in IMF height over time for each reconstruction method

**Figure 3:**
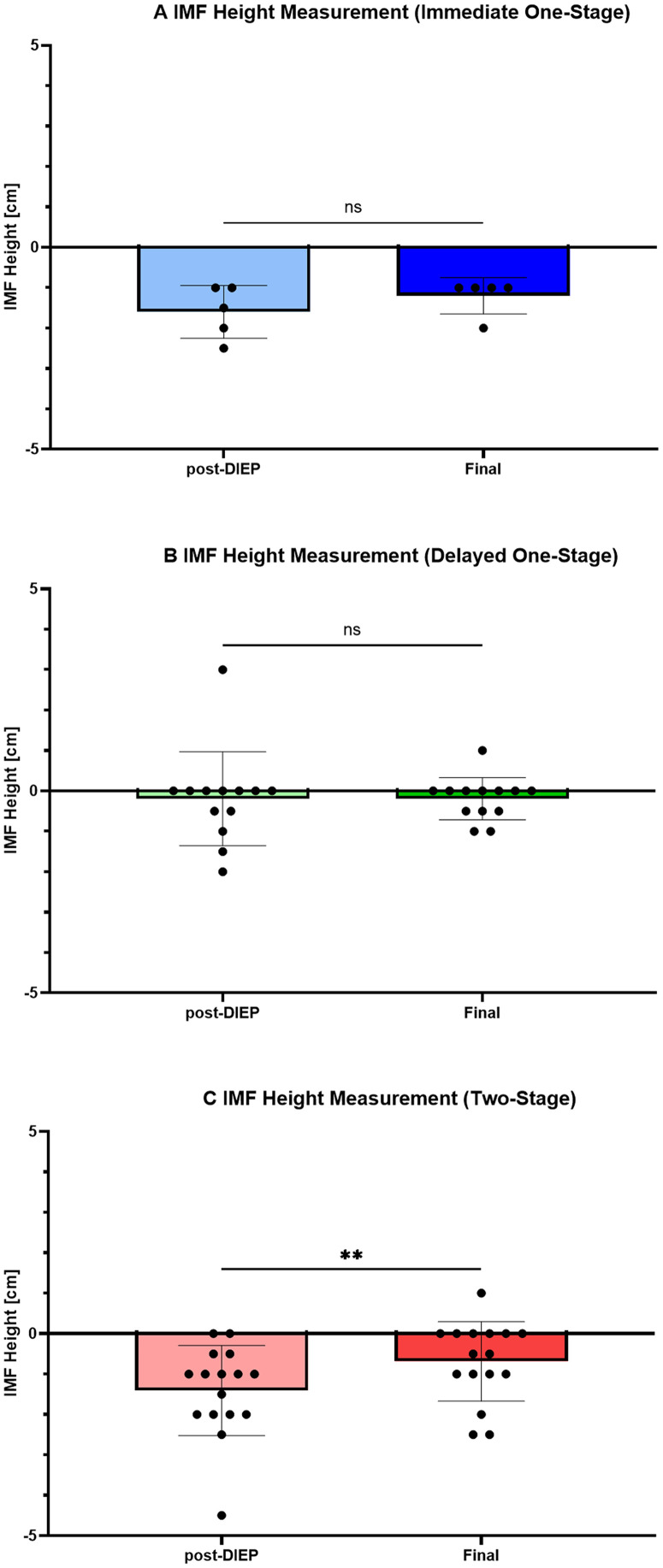
Change in IMF height over time for each reconstruction method. **A–C)** Results for immediate one-stage reconstruction (A), delayed one-stage reconstruction (B), and two-stage reconstruction (C). Wilcoxon signed-rank test; *p < 0.05, **p < 0.01, ***p < 0.001, ns, not significant.

The IMF for immediate one-stage reconstruction showed an average height of −1.60 ± 0.65 cm at post-DIEP and −1.20 ± 0.44 cm at final (*p* = 0.25; *n* = 5). The IMF for delayed one-stage reconstruction showed an average height of −0.19 ± 1.16 cm at post-DIEP and −0.19 ± 0.52 cm at final (*p* = 0.46; *n* = 13). The IMF for two-stage reconstruction showed an average height of −1.40 ± 1.11 cm at post-DIEP and −0.68 ± 0.98 cm at final (*p* = 0.0054; *n* = 16) (Figure 3A–C).

## Case presentation

### Immediate one-stage reconstruction

**Figure 4:**
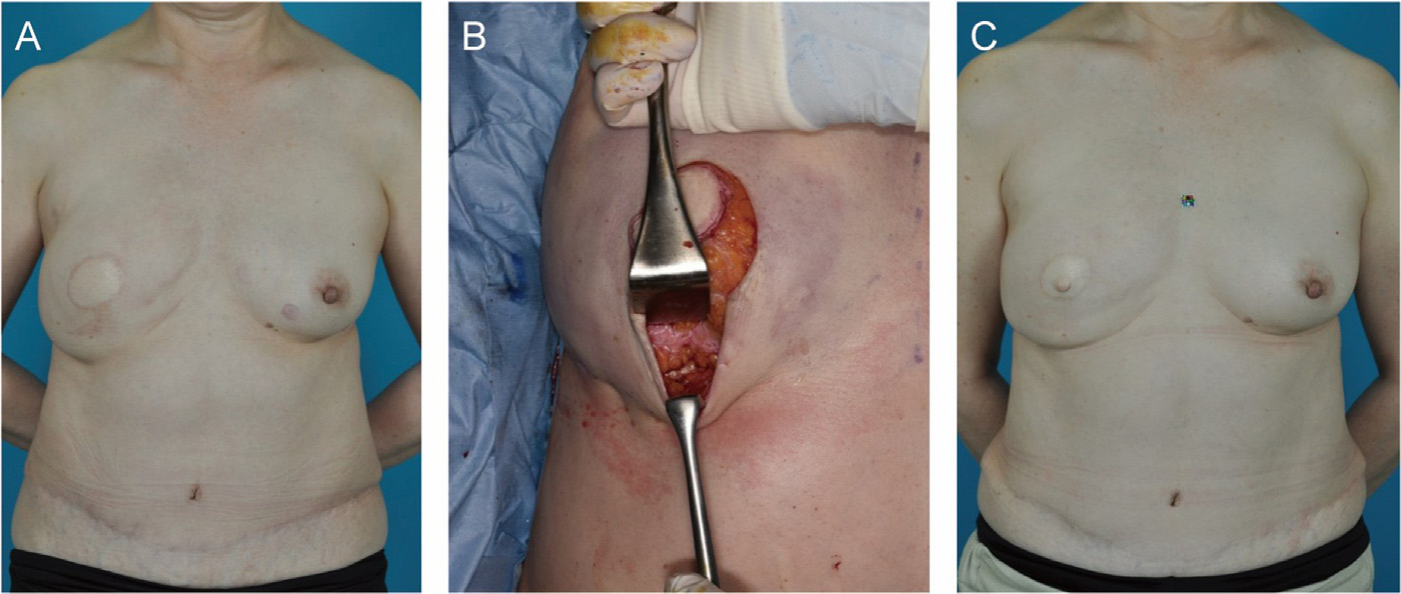
Patient after immediate one-stage breast reconstruction. **A)** IMF fixation in DIEP flap reconstruction. Six months after DIEP flap reconstruction, the affected side is 1.5 cm lower than the healthy side. **B)** Internal fixation is performed in the flap revision and NAC reconstruction surgery. **C)** Six months after the final IMF procedure, the IMF is fixed 1 cm lower.

In SSM and DIEP flap reconstruction, subcutaneous suture was performed for IMF fixation. The IMF was 1.5 cm lower on the affected side (Figure 4A), so an additional internal suture was made during flap revision and NAC reconstruction (Figure 4B). The final height of the IMF was 1 cm lower on the affected side (Figure 4C).

### Delayed one-stage reconstruction

**Figure 5:**
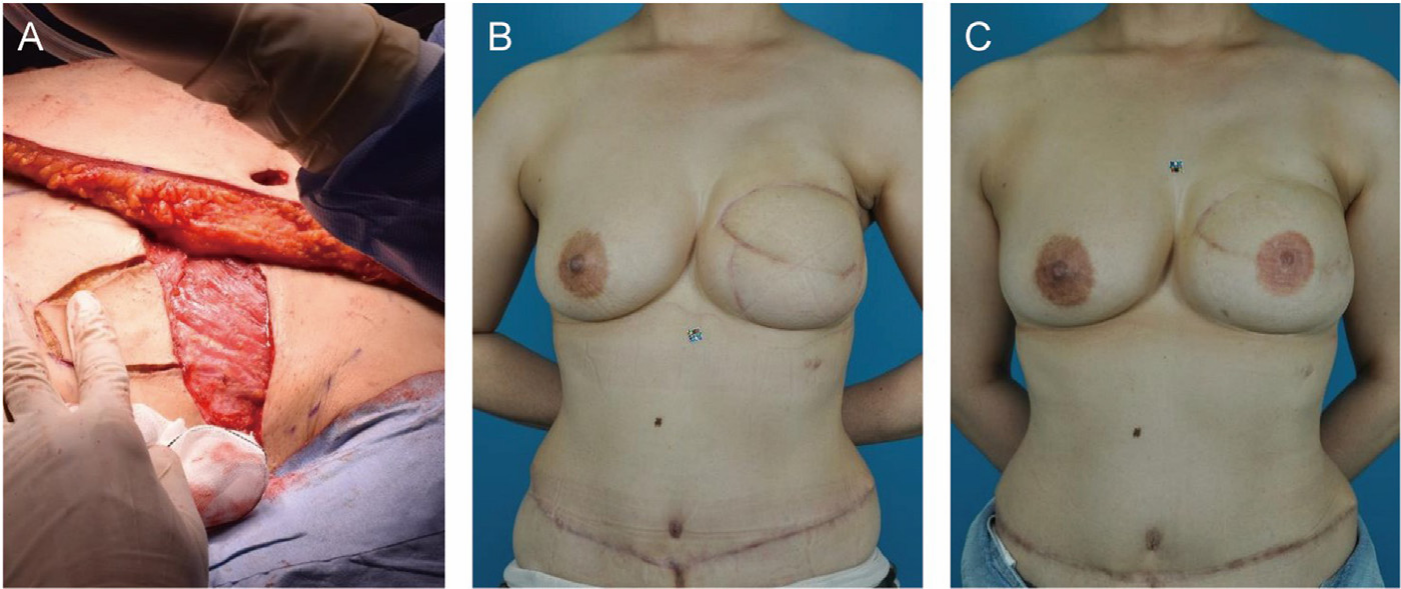
Patient after delayed one-stage breast reconstruction. **A)** The DIEP flap is fixed using the de-epithelialized method. **B)** Six months after DIEP flap reconstruction, the IMF is fixed within 0 cm, which does not require extra IMF fixation in NAC reconstruction surgery. **C)** Six months after NAC reconstruction surgery, the IMF is 0.5 cm lower than the healthy side.

For IMF fixation with DIEP flap reconstruction, the middle part of the mastectomy flap was de-epithelialized (Figure 5A). Six months after DIEP flap reconstruction, the affected side was fixed within 0 cm (Figure 5B). No flap revision or extra IMF fixation was required, so only NAC reconstruction was performed. In the final condition, the IMF was 0.5 cm lower on the affected side (Figure 5C).

### Two-stage reconstruction

**Figure 6:**
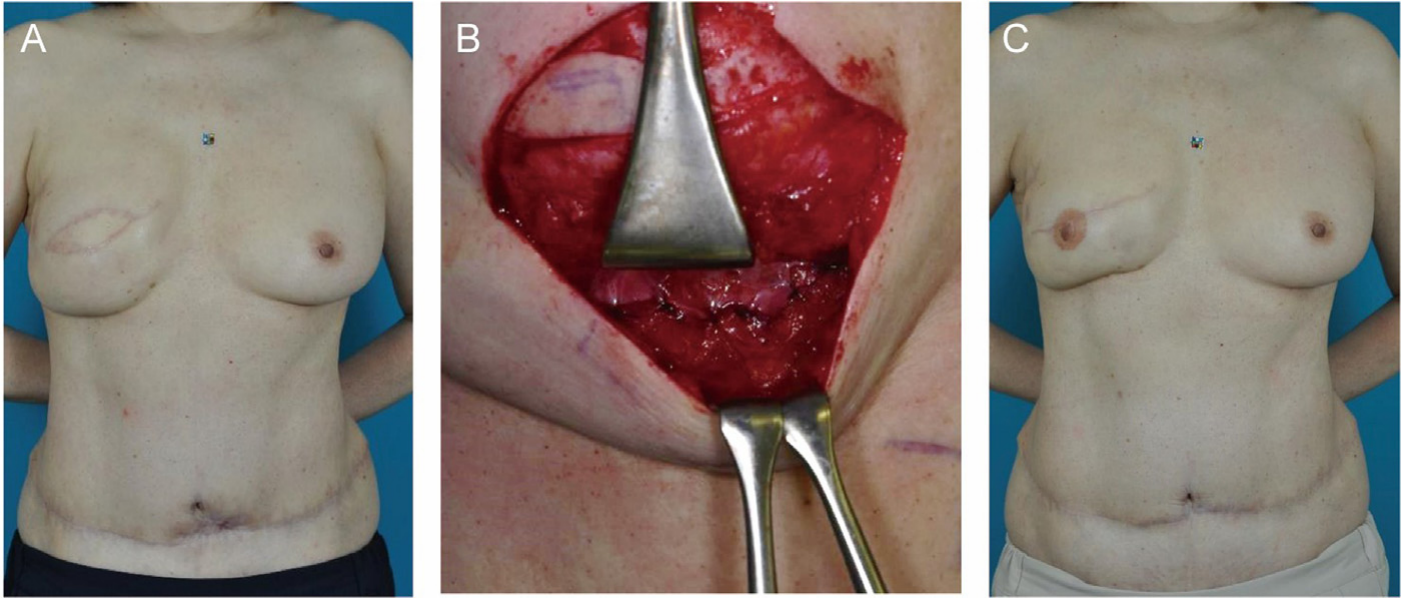
Patient after two-stage breast reconstruction. The IMF is fixed by tissue expander capsule repair. **A)** Six months after DIEP flap reconstruction, the IMF is 1 cm lower on the affected side than on the healthy side. **B)** The IMF procedure for flap revision and NAC reconstruction surgery involves additional repair of the capsule. **C)** Six months after the additional IMF procedure, the IMF is fixed within 0 cm.

After DIEP flap reconstruction, the IMF procedure involved tissue expander capsule repair. Six months after DIEP flap reconstruction, the IMF was 1 cm lower on the affected side (Figure 6A). For additional IMF fixation in flap revision surgery, pseudo-membrane was resected and capsule repair was performed (Figure 6B). In the final condition, the affected side was fixed within 0 cm (Figure 6C).

## Discussion

This study revealed that after unilateral DIEP flap reconstruction, the IMF on the affected side was significantly lower than the healthy side, particularly with immediate one-stage reconstruction or two-stage reconstruction, as compared to delayed one-stage reconstruction. After revision surgery, the IMF would be fixed within an acceptable range.

According to Blondeel,^16^ the main breast components are said to be the breast footprint, breast conus, skin envelope and NAC. The IMF is included in the breast footprint, making this essential to the breast structure. Various methods have been reported for fixation of the IMF, but few studies have compared IMF fixation methods. The attempt to create the IMF began with external fixation,^5^^,^^17^ but the internal approach has gained popularity^6^^,^^7^ because of the avoidance of additional scarring.

With the immediate one-stage reconstruction, the IMF is often said to be preserved. However, the IMF structure is destroyed in some cases. Even if we think the IMF is preserved during mastectomy, we perform internal fixation in immediate one-stage reconstruction. Indeed, all five cases with immediate one-stage reconstruction in this series required IMF fixation in both DIEP flap reconstruction and flap revision surgery. In immediate one-stage reconstruction, compared to other groups, the mastectomy procedure was mainly SSM which may suggest the initial preservation of the IMF might interact with the IMF fixation results. The combination of the internal fixation and the oppositional pull due to the abdominal donor site closure could be responsible for these relatively poor results of IMF height in immediate one-stage reconstruction. For mastectomy the presence of SSM was relatively high in immediate one-stage reconstruction compared to delayed one-stage or two-stage reconstruction which could be responsible for the poor results. In delayed one-stage reconstruction cases or two-stage reconstruction cases, confirming the presence of a structurally intact IMF is difficult and we often perform an IMF fixation procedure in DIEP flap breast reconstruction. For cases of two-stage reconstruction, capsule repair was selected for IMF definition. This method^8^ offers strength because no additional scars are created on the surface, and a smooth IMF can be created compared with the subcutaneous sutures with indentations. To compare IMF fixation methods, we conducted this retrospective study of breast reconstruction cases requiring IMF fixation.

The results showed that with delayed one-stage reconstruction, the de-epithelialized mastectomy flap^9^ appeared optimal in terms of creating the IMF. This surgical method offers superior cosmetic results due to only having a single visible scar and allowing anatomical recreation of the IMF, yielding better reconstruction of the subunits of the breast. However, due to the unacceptable upward displacement seen in two NSM cases, the NAC was excised and the de-epithelialized method was applied. Delayed one-stage reconstruction demands a skin island to replicate the ptosis and needs strong tissue to support the DIEP flap but represents a good option considering the initial results of IMF reconstruction. In addition, delayed one-stage cases involved large proportion of patients receiving radiation therapy prior to DIEP flap reconstruction, this may explain the IMF retaining effect due to the skin irradiation making the de-epithelialized method more stable.

The results suggest that IMF fixation alone in DIEP reconstruction surgery would be insufficient for some cases, as all cases with immediate one-stage reconstruction required additional fixation in the flap revision surgery. More than half of delayed one-stage cases and two-stage reconstructions also required extra IMF procedures. After the required procedures, the average IMF height ranged from −1.2 cm to −0.19 cm compared with the healthy side, which could be considered an acceptable result.

According to anatomical studies, the IMF is a layered membrane-like structure originating from the 5th to the 7th rib.^18^, ^19^, ^20^ Considering each method of IMF reconstruction, internal fixation to the chest wall, which is mainly used in immediate one-stage reconstruction^5^, ^6^, ^7^ could be recognized as a “spot” fixation. The de-epithelialized mastectomy flap performed in delayed one-stage cases could mimic the direction of supporting force for the real IMF by fixing the IMF by “plane”.^9^ With the capsule suturing method^8^ mainly used in two-stage reconstructions, the direction of lift for the IMF would be vertical, differing slightly from the direction of the real IMF and supporting the IMF by “line”. Reconstructing the structure of the anatomic IMF is difficult because of the multi-layered loose connected tissue. With the IMF reconstruction methods used here, the de-epithelialized method fixing the DIEP flap by “plane” would be a good option in terms of both direction of lift and the retaining ability shown in this study. Some studies have focused on how to set the IMF in the breast reconstructed with fat graft,^21^^,^^22^ but few have focused on the IMF from DIEP flap reconstruction. This study compared multiple types of IMF reconstruction and thus may provide useful insights into IMF definition using autologous flap reconstruction.

Several limitations to this study must be acknowledged. This was a single-center retrospective study with a small sample size. Due to the study design, there are confounders such as radiotherapy, mastectomy method or the heterogeneity in flap revision surgery which prevent the definitive conclusion. The disparity of radiation status in immediate one-stage reconstruction (0%), delayed one-stage reconstruction (46%) and two-stage reconstruction (7%) would be a confounding factor. Variation in the mastectomy method, for example for SSM, immediate one-stage reconstruction (100%), delayed one-stage reconstruction (8%) and two-stage reconstruction (43%) would be another confounding factor. The IMF fixation techniques during the flap revision surgery varied in the cohort, with internal fixation being the most common, though capsule repair and others were also employed in some cases. Acknowledgement of heterogeneity in fixation techniques during flap revision surgery is needed for interpretation of our results. The selection of the IMF fixation method was based on the reconstruction timing; it is yet unclear whether the IMF fixation method itself is responsible for the improved IMF results. Prospective studies with standardized protocols are required to isolate these variables. Patients with severe breast ptosis were excluded, so cases were weighted toward relatively small breasts. Although trends are seen in IMF reconstruction procedures, this analysis grouped cases solely by reconstruction methods, so we cannot say that surgical procedures were totally controlled for in the present study.

## Conclusion

After DIEP flap reconstruction, regardless of the reconstruction method and IMF procedure, the reconstructed IMF demonstrated an inferior displacement on the affected side than on the healthy side. However, by fixing the IMF in the flap revision surgery after DIEP flap reconstruction, the average IMF height ranged from −1.2 cm to −0.19 cm compared to the healthy side. Particularly with delayed one-stage reconstruction, the de-epithelialized mastectomy flap was used for the IMF procedure and achieved good results. This retrospective study compared IMF height between reconstruction methods, providing potential insights into future IMF recreation. Further studies are required to compare long-term results of IMF fixation in breast reconstruction using autologous tissues.

## Author contribution

**H.M.** designed the study, the main conceptual ideas, and the proof outline. **T.K., N.U., S.K., K.M., N.H. and H.M.** collected the data. **T.K. and H.M.** aided in interpreting the results and drafted the manuscript. **H.M., K.K., N.U. and K.T.** supervised the project. **T.K.** wrote the manuscript with support from **H.M.** and **K.T.** All authors discussed the results and commented on the manuscript.

## Funding

None.

## Ethical approval

This retrospective study was conducted at Institute of Science Tokyo Hospital, Tokyo, Japan and conducted with the accordance within the declaration of Helsinki. The institutional review board of the Institute of Science Tokyo Hospital (IRB-number M2000-2251 on September 28th, 2015) approved of this study.

## Declaration of competing interest

None.

## Appendix Supplementary materials

Image, application 1
